# Supplementary material for: Essential Tremor Disrupts Rhythmic Brain Networks During Naturalistic Movement
Source: Neurobiol Dis. Author manuscript; Available in PMC 2025 Apr 3. (PMC7617547; doi:10.1016/j.nbd.2025.106858)
Supplement: Supplementary Information [file EMS204252-supplement-Supplementary_Information.docx]

# Supplementary Figures

## Supplementary Figure 1 – Analysis of Essential Tremor Properties

| 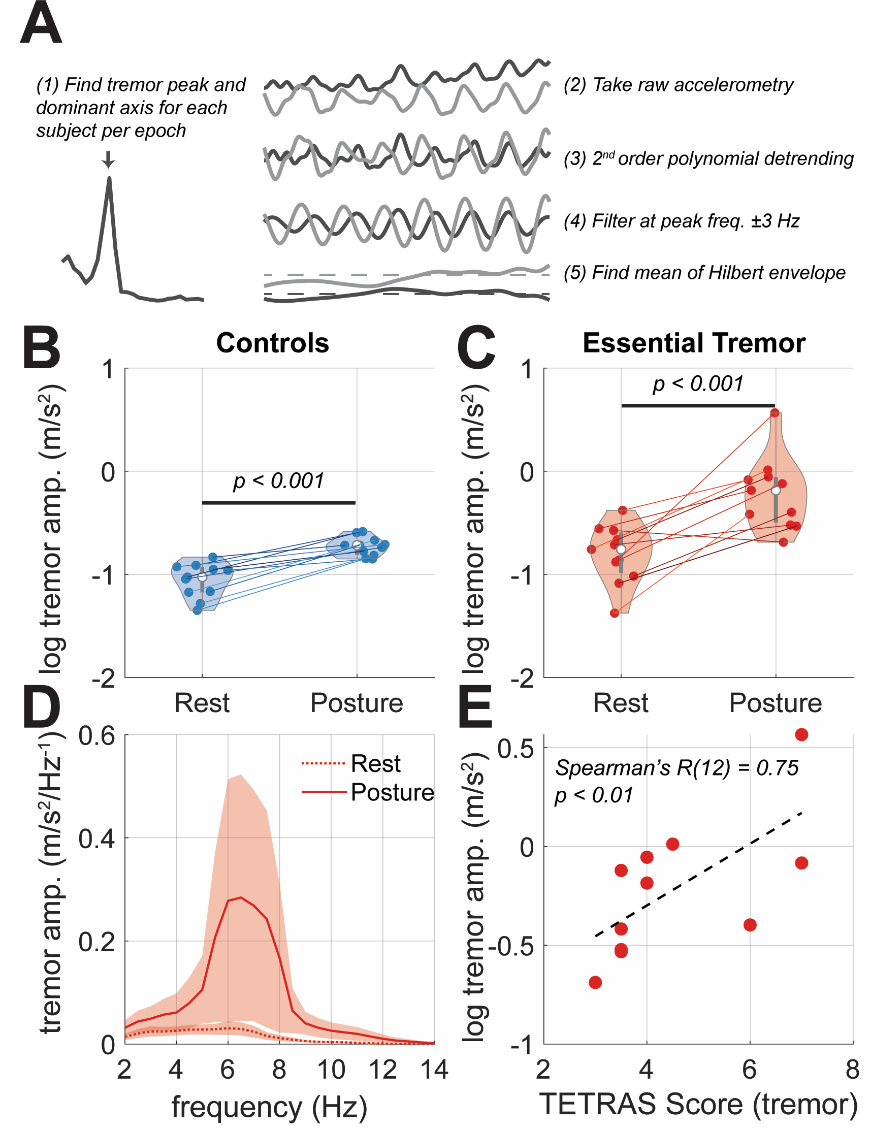 |
| --- |
| Supplementary Figure 1 – **Analysis of tremor dynamics during steady state recordings at rest and posture.** Violin plots indicate the distribution of tremor amplitudes over either the control (blue) or ET (red) cohorts), with lines linking individuals between rest and posture. Statistics indicate outcomes of paired t-tests. **(A)** Illustration of processing pipeline to compute the tremor amplitude. **(B)** Control subjects show a small increase in physiological tremor from rest to posture. **(C)** As expected, ET subjects show a significant increase in tremor at posture. **(D)** The average tremor peak frequency was around 6 Hz, increasing slightly during posture trials. Bounds give the SEM across ET patients. **(E)** Measures of tremor amplitude at posture positively correlate with clinical TETRAS assessment of tremor severity. A regression line is shown alongside, Spearman’s correlation coefficient. |

## Supplementary Figure 2 –Effects of Experimental Conditions on Tremor

| 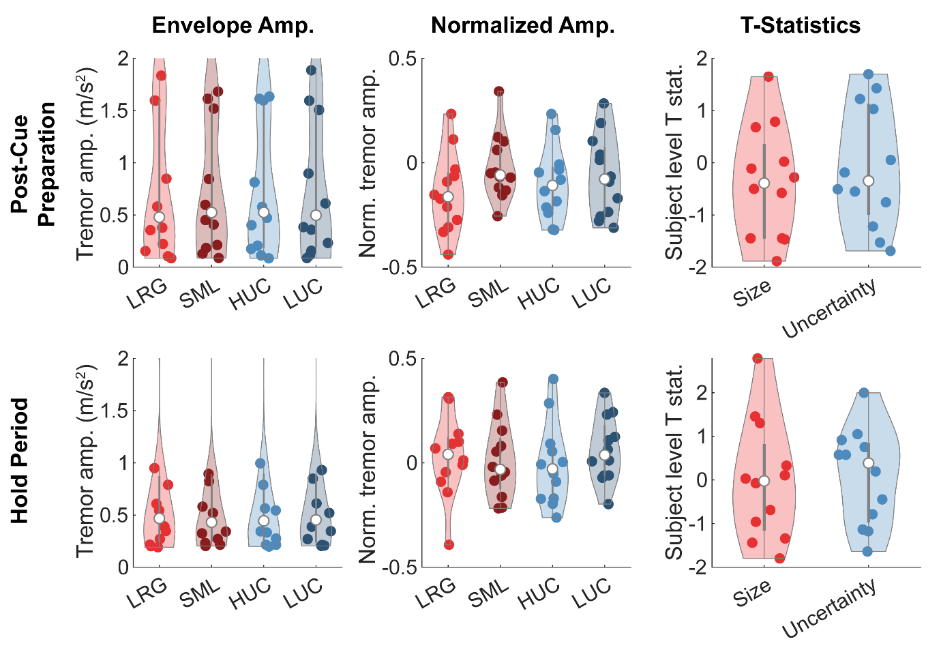 |
| --- |
| Supplementary Information 2 – **Analysis of modulation of tremor amplitude by task conditions.** Tremor amplitude was estimated as in Figure 2A. We divided trials by the 2x2 conditions modulating either target size (SML or LRG) and the uncertainty of directional cues (HUC or LUC). (1^st^ Column) The violin plots here show the distribution of tremor power across the conditions. Each point represents the mean of an individual subject. (2^nd^ Column) The distributions of tremor amplitude normalized by the subject level mean and standard deviation. (3^rd^ Column) A plot of the subject level T-statistics comparing each of the experimental conditions. No significant differences between conditions were found. |

## Supplementary Figure 3 - Source level distribution of movement responsive beta power

| 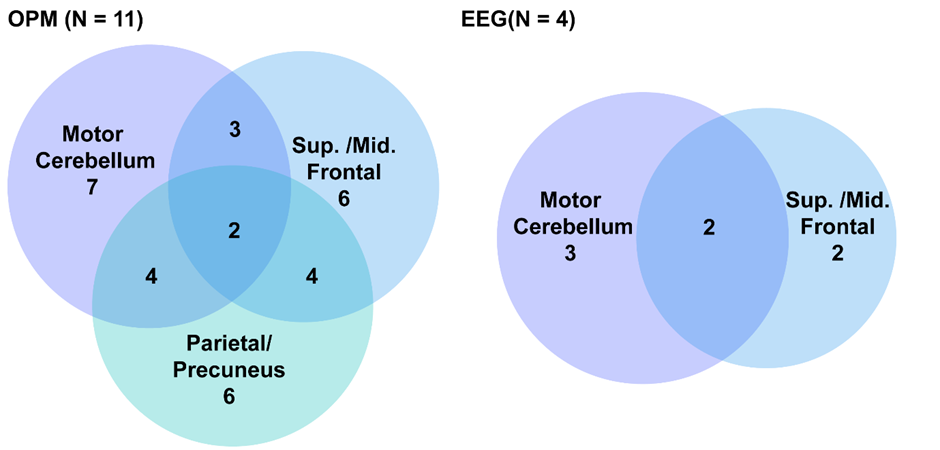 |
| --- |
| Supplementary Figure 3 – Venn diagrams of the total number of patients exhibiting a peak (over 10% of voxels within region >85^th^ percentile of the total source map) in the auxiliary DICS map of sources coherent with the tremor accelerometery. The cSMA was regressed from these subject to reveal coherent sources outside of the main dominant source found across the majority of patients studied.  . |

## Supplementary Figure 4 - Source level distribution of movement responsive beta power

| 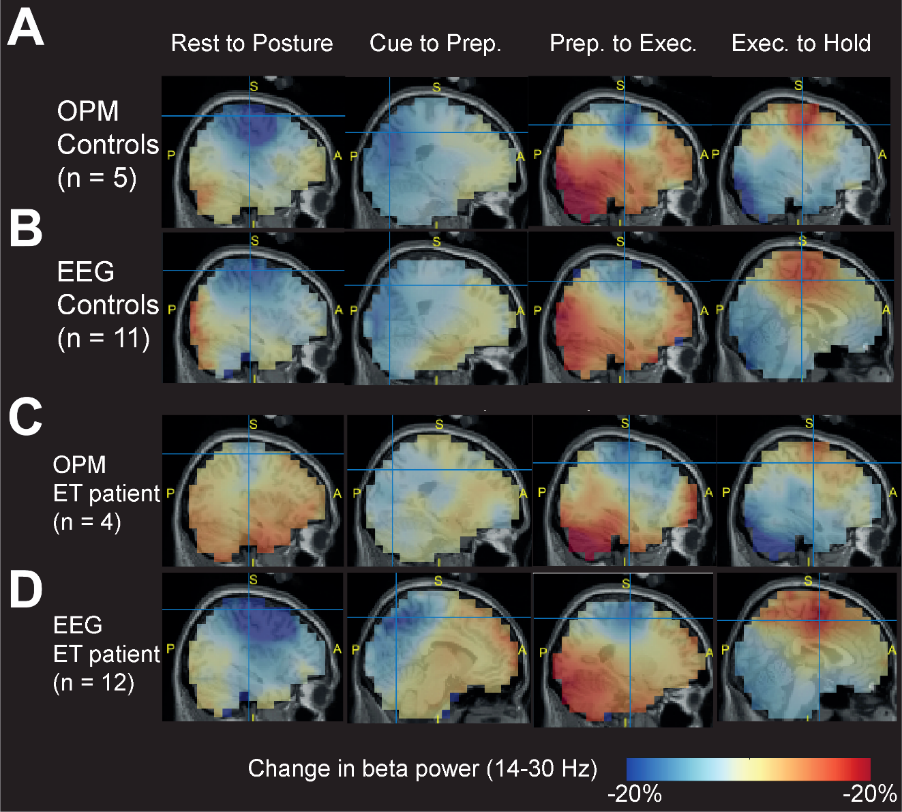 |
| --- |
| Supplementary Figure 4 – **Source level distribution of 14-30 Hz beta power.** Beta power was localized using a DICS beamformer. Differences in power relative to transitions during the reaching task were computed. Source images were normalized at a subject level before averaging. **(A)** Data for age-matched controls recorded with OPMs; **(B)** age-matched controls recorded with high density EEG; **(C)** Essential Tremor patients recorded with OPMs; **(C)** Essential Tremor patients recorded with high density EEG. |

## Supplementary Figure 5 - Spectrograms for Virtual Electrodes in Control Subjects

| 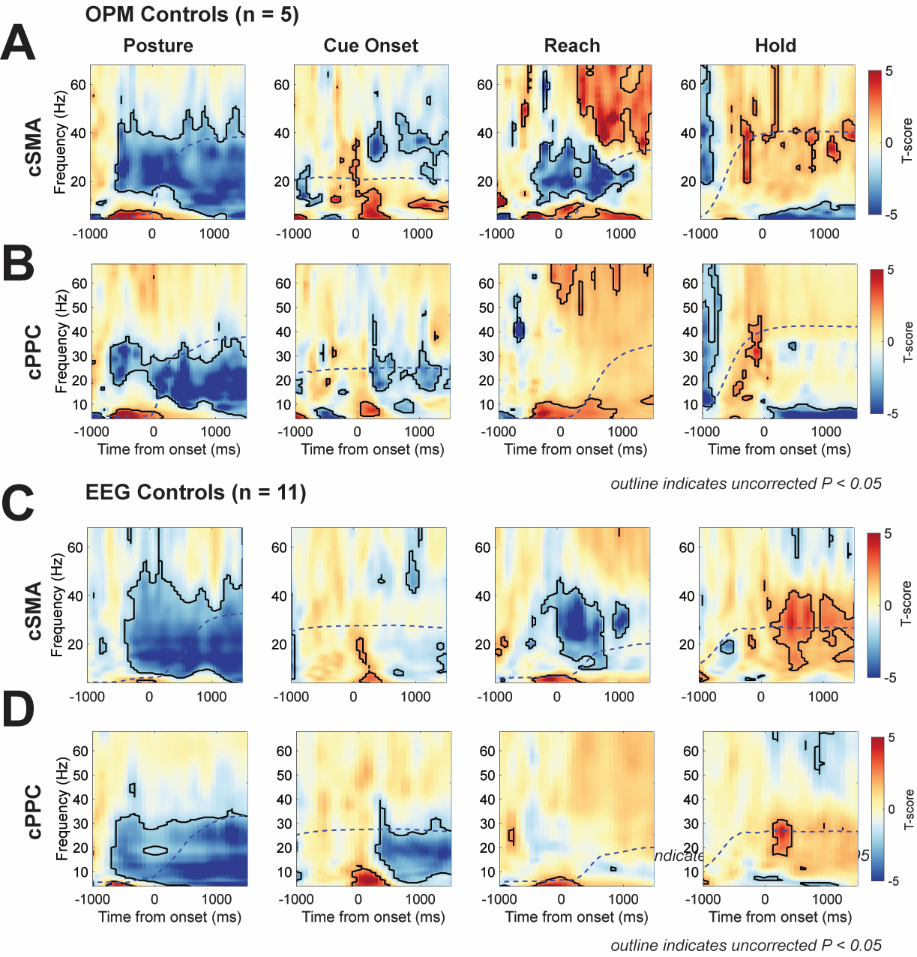 |
| --- |
| Supplementary Figure 5 - **Time-frequency spectrograms of time locked activity in either the cSMA or cPPC, in both OPM and EEG data in control subjects.** Panels show the group level t-statistics of time-frequency spectrograms constructed from virtual channels, compared to baseline. Bold outlines indicate thresholding on the critical T (P < 0.05; uncorrected). Dashed lines overlaid indicate the group averaged movement trace. **(A)** Spectrograms of OPM derived cSMA activity for time locked activity at onset of postural hold (1^st^ column); onset of directional cues (2^nd^ column); onset of reach (3^rd^ column); and establishment of the hold period (4^th^ column). **(B)** Same as (A), but for cPPC activity. **(C-D)** Same as A-B, but for EEG data. |

## Supplementary Figure 6 - Spectrograms for Virtual Electrodes in Essential Tremor Subjects

| 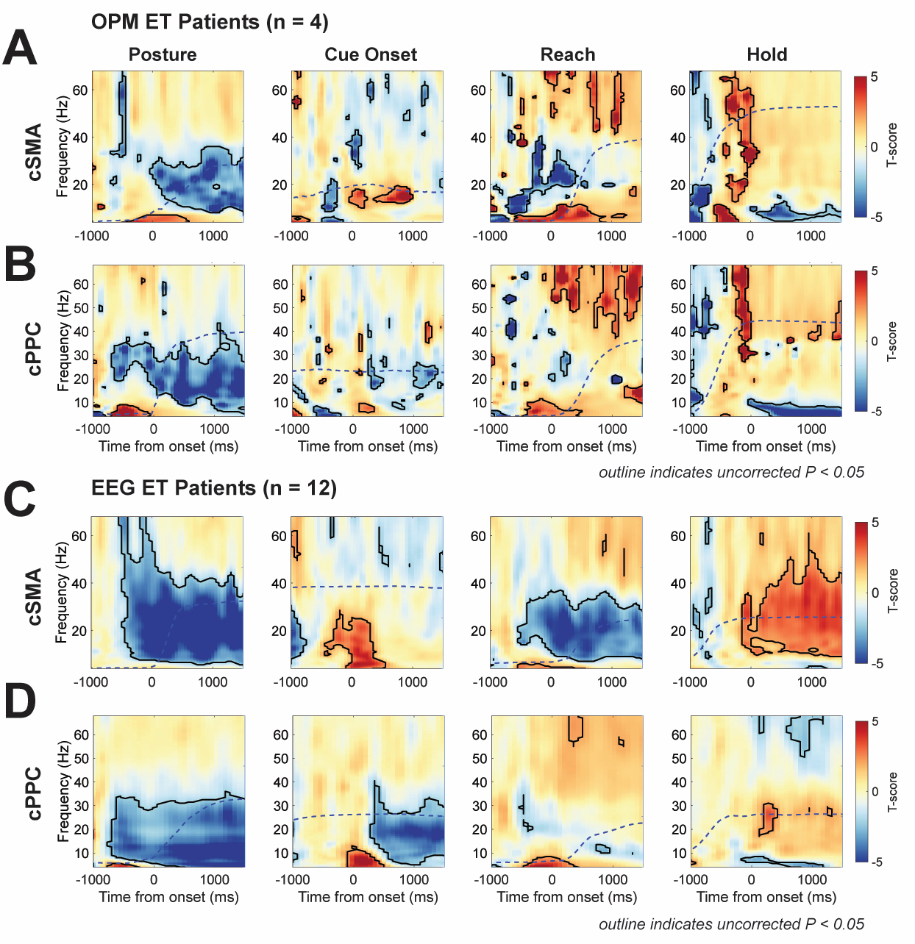 |
| --- |
| Supplementary Figure 6 – **Time-frequency spectrograms of time locked activity in either the cSMA or cPPC, in both OPM and EEG data in ET patients.** Panels show the group level t-statistics of time-frequency spectrograms constructed from virtual channels, compared to baseline. Bold outlines indicate thresholding on the critical T (P < 0.05; uncorrected). Dashed lines overlaid indicate the group averaged movement trace. **(A)** Spectrograms of OPM derived cSMA activity for time locked activity at onset of postural hold (1^st^ column); onset of directional cues (2^nd^ column); onset of reach (3^rd^ column); and establishment of the hold period (4^th^ column). **(B)** Same as (A), but for cPPC activity. **(C-D)** Same as A-B, but for EEG data. |

## Supplementary Figure 7 – Comparison Of Whole Brain, Motor Responsive Latent Networks Between Controls and ET Patients Using OPM Data

| 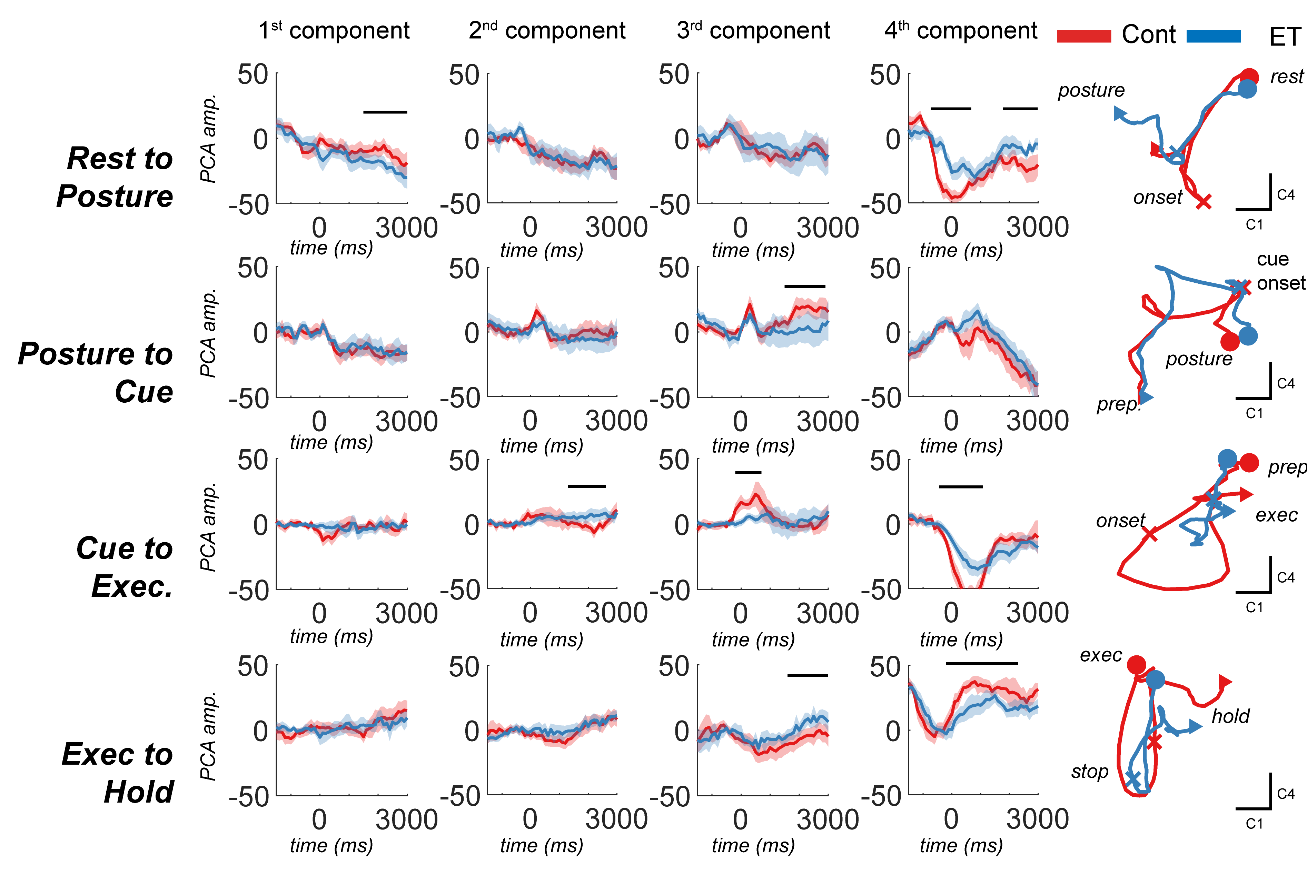 |
| --- |
| Supplementary Figure 7 –**Visualization of whole brain, motor responsive, latent dynamics and a comparison between controls and patients with ET recorded with OPM.** Components were computed using tfPCA applied to the group averaged EEG data (Figure 5). These coefficients were then used to project data to trial-level latent dynamics that could be used to explore differences between controls and ET patients. **(A)** The latent dynamics exhibited during the postural hold for each component (columns) are indicated for ET (blue) and controls (red) separately. Bars and associated P-values show the outcome of cluster permutation statistics between the two experimental groups. **(B-D)** Same as (A) but for cue presentation, reach execution, and the sustained hold. **(E-H)** Plots of the 2D latent trajectories (components 1 and 4, only) indicate highly correlated dynamics between controls and ETs with quantitative differences in the weighting of the components such as increased engagement of the prefrontal beta network in ET subjects (4^th^ component, apparent in plot G). |

## Supplementary Figure 8 – Comparison Of Whole Brain, Motor Responsive Latent Networks Between Controls and ET Patients Using OPM Data

| 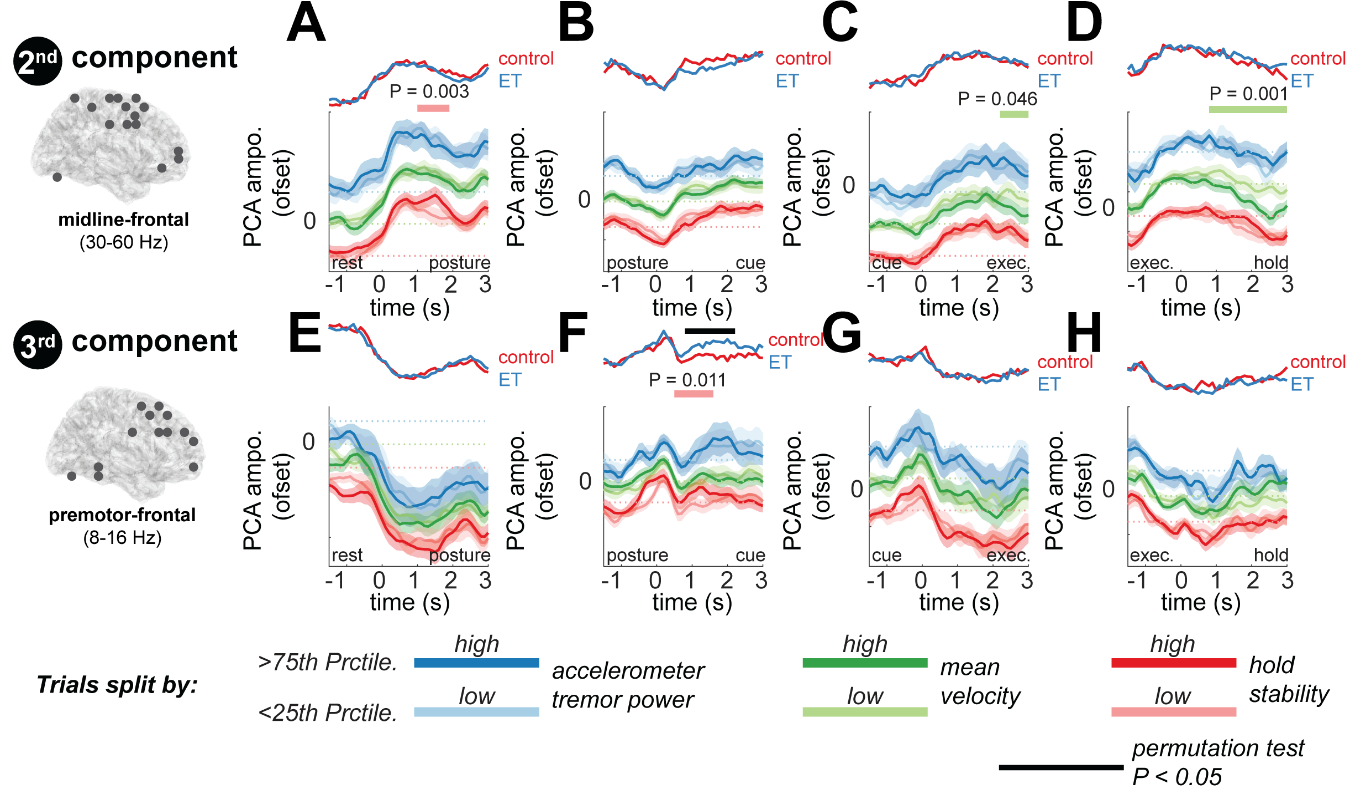 |
| --- |
| Supplementary Figure 8 –**Visualization of whole brain, motor responsive, latent dynamics and a comparison between controls and patients with ET recorded with OPM.** Components were computed using tfPCA applied to the group averaged EEG data (Figure 5). These coefficients were then used to project data to trial-level latent dynamics that could be used to explore differences between controls and ET patients. **(A)** The latent dynamics exhibited during the postural hold for each component (columns) are indicated for ET (blue) and controls (red) separately. Bars and associated P-values show the outcome of cluster permutation statistics between the two experimental groups. **(B-D)** Same as (A) but for cue presentation, reach execution, and the sustained hold. **(E-H)** Plots of the 2D latent trajectories (components 1 and 4, only) indicate highly correlated dynamics between controls and ETs with quantitative differences in the weighting of the components such as increased engagement of the prefrontal beta network in ET subjects (4^th^ component, apparent in plot G). |

## Supplementary Figure 9 – Convolutional Neural Network for Movement Detection

| 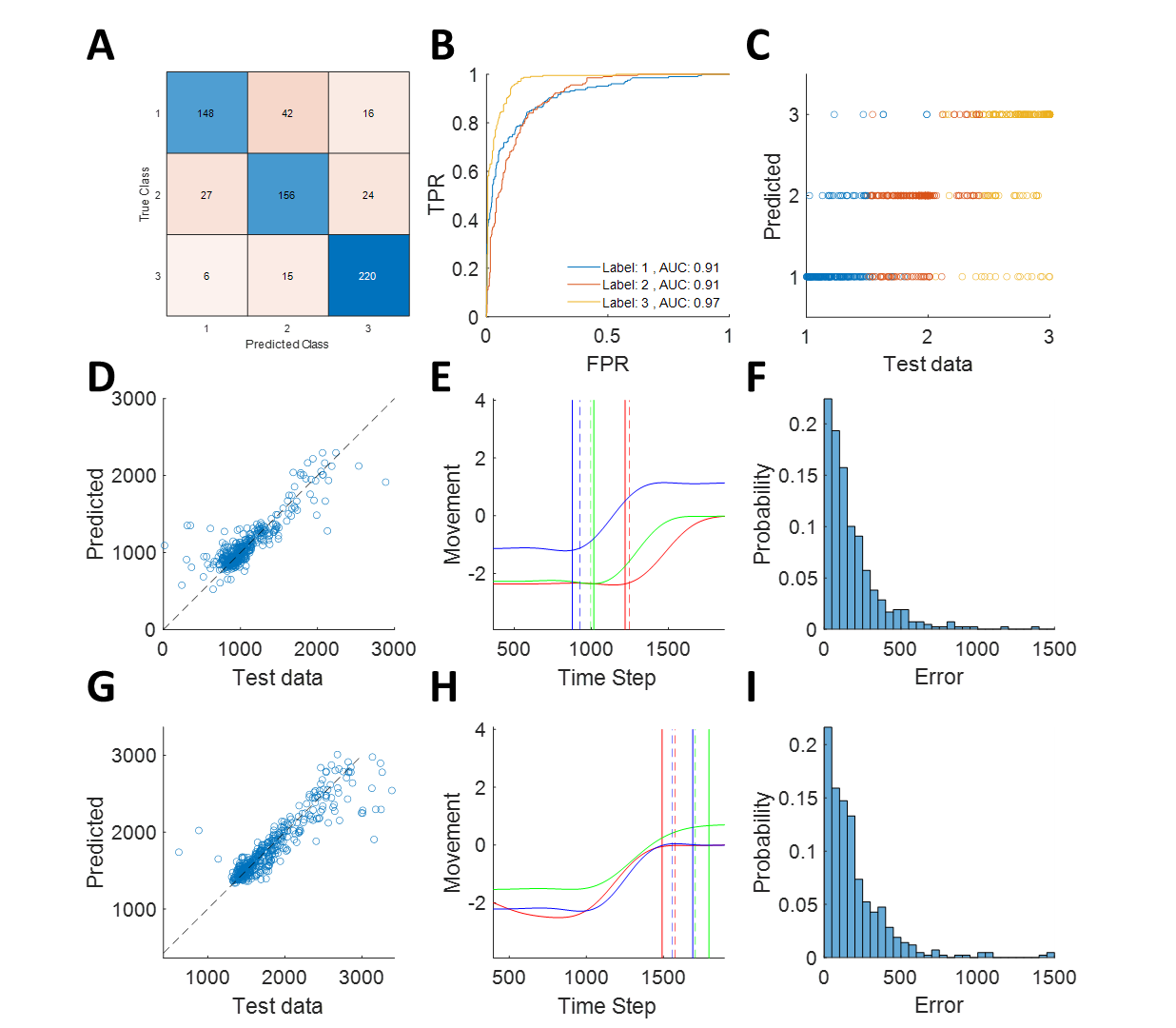 |
| --- |
| Supplementary Figure 9 – **Validation of convolutional neural network model for time locking of reaching movement.** Plots indicate results examining the performance of a CNN based classifier/regression for reach quality (A-C); reach onset (exec.; D-F) and termination (hold; G-I). Training data was provided by manual labelling of data from three separate assessors on ~30% of the data. **(A)** Confusion matrix of recording quality class: (1) is optimal, (2) sub-optimal, (3) is rejected. See Supplementary Information II for criteria. **(B)** Receiver operating characteristics for reach quality classifier, with each of the three data quality labels. **(C)** Scattergram of predicted vs test data. **(D)** Same as (C) but for prediction of reach onsets. **(E)** Example plot of three predicted (bold) vs manually marked (dashed) reach onsets. **(F)** Histogram of the model error (\|predicted-manual\|, ms). **(G-I)** Same as D-F, but for reach holds. |

# Supplementary Information

## Supplementary Information I – Clinical Table: EEG Patients

| Subject | Age | Gender | TETRAS Performance | TETRAS Upper Limb Tremor (R/L) | TETRAS Head Tremor | Medication |
| --- | --- | --- | --- | --- | --- | --- |
| 1 | 53 | M | 12.5 | 3.5/3 | 0 | Gabapentin 600 mg; Primidone 500mg; Amitriptyline 75 mg |
| 2 | 73 | M | 15.5 | 3/4 | 1 | Primidone 150mg; Propranolol 40mg; L-Thyrpx 100mg; Ramipril 2.5 mg |
| 3 | 68 | M | 23 | 4/6 | 0 | Propranolol 40mg; Amlodipine 5mg; |
| 4 | 59 | M | 38 | 6.5/8 | 1 | Propranolol 80mg; Amlodipine 5mg; Insulin Glargine + Lispro; Clopidogrel 75mg; Simva 20mg; Ramipril 10mg |
| 5 | 19 | F | 7.5 | 1/3.5 | 0 | None |
| 6 | 19 | M | 11 | 3/3 | 0 | None |
| 7 | 58 | F | 15.5 | 3/4 | 1 | Propranolol 10mg; Escitalopram 15mg |
| 8 | 24 | F | 9 | 3.5/3 | 0 | None |
| 9 | 21 | F | 17 | 3.5/7 | 0 | Propranolol 10mg |
| 10 | 61 | M | 27.5 | 7/6 | 2 | Propranolol 40mg; Liskantin 250mg |
| 11 | 42 | M | 14 | 4.5/4.5 | 0 | None |
| 12 | 64 | F | 13 | 3/3.5 | 1 | Propranolol 20mg |

## Supplementary Information II – Clinical Table: OPM Patients

| Subject | Age | Gender | TETRAS Performance | TETRAS Upper Limb Tremor (R/L) | TETRAS Head Tremor | Medication |
| --- | --- | --- | --- | --- | --- | --- |
| 1 | 67 | F | 19 | 5/4 | 0 | None |
| 2 | 44 | F | 25 | 7/7 | 0 | None |
| 3 | 66 | M | 17 | 6/6 | 0 | Amlopidine, Pravastatin, NiC for tremor |
| 4 | 25 | F | 17 | 3/6 |  | Primidone 25 mg |

## Supplementary Information III – Analysis of Reproducibility of Latent Dynamics

**Table 1 –** Analysis of Correlation Between Latent Dynamics in Controls and ET Recordings

|  | **EEG** | | | | |
| --- | --- | --- | --- | --- | --- |
|  | **Comp 1** | **Comp 2** | **Comp 3** | **Comp 4** | *Average* |
| **Posture** | 0.96 | 0.98 | 0.98 | 0.94 | *0.96* |
| **Cue** | 0.91 | 0.95 | 0.68 | 0.86 | *0.85* |
| **Reach** | 0.90 | 0.98 | 0.91 | 0.95 | *0.94* |
| **Hold** | 0.88 | 0.90 | 0.88 | 0.98 | *0.91* |
| **Average** | *0.91* | *0.95* | *0.86* | *0.93* | ***0.91*** |
|  | **OPM** | | | | |
|  | **Comp 1** | **Comp 2** | **Comp 3** | **Comp 4** | *Average* |
| **Posture** | 0.90 | 0.90 | 0.83 | 0.88 | *0.88* |
| **Cue** | 0.96 | 0.68 | 0.42 | 0.97 | *0.76* |
| **Reach** | 0.84 | 0.85 | 0.89 | 0.94 | *0.88* |
| **Hold** | 0.87 | 0.90 | 0.76 | 0.85 | *0.85* |
| *Average* | *0.89* | *0.83* | *0.73* | *0.91* | ***0.84*** |

**Table 2 –** Analysis of Correlation Between Latent Dynamics in OPM and EEG

| Analysis of Correlation Between Latent Dynamics in OPM and EEG (Pearson’s Correlation Coefficient R) | | | | | |
| --- | --- | --- | --- | --- | --- |
|  | **Component 1** | **Component 2** | **Component 3** | **Component 4** | *Average* |
| **Posture** | 0.93 | 0.72 | 0.65 | 0.93 | *0.81* |
| **Cue** | 0.94 | 0.62 | 0.47 | 0.99 | *0.76* |
| **Reach** | 0.97 | -0.03 | 0.46 | 0.94 | *0.59* |
| **Hold** | 0.93 | 0.56 | 0.65 | 0.87 | *0.75* |
| *Average* | *0.94* | *0.47* | *0.56* | *0.93* | ***0.73*** |

## Supplementary Information IV – Details of the Delayed Reach-to-Target Task

To probe the interaction between tremor and voluntary motor control, participants performed a delayed reach to target task, making whole limb reaches to mimic naturalistic movement. The task (Figure 1A) required participants to adopt a 90-degree upper arm elevation against gravity with flexed elbow posture and then, from this position, make centre-out reaches and hold their finger fixed to “pop” target balloons. The task sequence was: (a) 3 s eyes-open rest, (b) 3 s postural (as above) hold, (c) 2.5 ±1 s presentation of movement cues, given as arrows of varying length, (d) appearance of a GO cue to initiate a reach, to one of eight targets, (e) 1.5 s of sustained hold required to “pop” the balloon. A point was scored if the correct balloon was popped (as predicted from the dispersion of the arrows). A maximum of 4 s was given to make the reach and hold. In addition, 30 s of rest and postural hold (i.e., the posture described above) was recorded for each block. We recorded 6 sessions of 24 reaches (~12 minutes each), yielding an average of 144 reaches per participant.

The task followed a 2x2 design manipulating cue uncertainty and target size. Cue uncertainty was set to be low or high (LUC and HUC, respectively), by changing the dispersion of arrows (see Supplementary Information III for details) titrated to success rates of 85% and 15%, respectively. The size of the targets (i.e., the balloons) were either small or large (SML and LRG, respectively). For EEG recordings the task was presented on an LCD monitor (30 x 53 cm, 60 Hz, SML/LRG diameters: 1.4/3.6 cm) placed at arm’s length from the participant. For OPM recordings, the task was presented via a projector to a screen (42 x 70 cm, 60 Hz, SML/LRG diameters: 2/5 cm).

Let *D* be the set of cardinal directions, where *D = {N, NE, E, SE, S, SW, W, NW}*. A direction *d* is selected from *D*, where *d* ∈ *D*. We generate a set of N random phases (expressed in radians):

$$\theta_{n}=\bar{\theta}+\sigma\epsilon_{n}, n=1,2,\ldots,N$$

where $\bar{\theta}$and $\sigma$ represent the chosen angle and dispersion (uncertainty) of the movement cues, and $\epsilon_{n}$ is a random variable sampled from a standard normal distribution, $\epsilon_{n}\sim N(0,1)$. The direction of each arrow is then wrapped around the unit circle: $\phi_{n}=\arg\left( e^{i\theta_{n}} \right).$The arrow directions are then determined by computing the normalized histogram for bins with edges corresponding to the cardinal directions, to yield a scalar for each arrow. We used $\sigma$= 0.98 for low uncertainty trials, and $\sigma$= 1.64 for high uncertainty trials, yielding 85% and 15% average success rates in piloting with healthy controls.

## Supplementary Information V – Details of the Automatic Timelocking and Criteria for Reach Quality Marking

A convolutional neural network (CNN) was trained to detect movement onsets and endpoints from a subset of triaxial accelerometer, and 3D motion tracking data that was manually marked by three separate researchers. The CNN consisted of two convolutional layers (1^st^ dimension [6 x 8]; and 2^nd^ dimension [6 x 16]), each interleaved with batch normalisation, ReLU activations, and average pooling (stride of two). A fully connected layer was then used to combine activations into a regression layer with a single output. Three models were trained separately for movement onsets, endpoints, and the reach quality (see below). 15% of the data was held out for model validation.

Criteria for manual marking of reach quality are detailed below:

*Grade 1 – Optimal Reach*

- A stable baseline is present prior to movement, with little oscillation or prepotent movement.
- The reach may be initiated before the movement cue (i.e., t < 0) if baseline is stable.
- The reach itself is smooth, with a clear stereotyped trajectory.
- The hold period is stable and lasts greater than 2 seconds, with no clear drifts or repositioning of the hand.

*Grade 2 – Suboptimal Reach*

- A baseline period is apparent, although some slow drifts in posture, or small prepotent movements are present.
- The reach may be initiated before the movement cue (i.e., t < 0) if the baseline is stable.
- The reach is jerky, or non-smooth containing mid-reach corrections and changes in velocity.
- The hold period is apparent and last for a duration >= 2 seconds. The hold period may contain slow drifts, or small corrective movements.

*Grade 3 – Rejected Reach*

- The reach is slow (> 4 seconds).
- The reach is jerky/non-smooth.
- There is no clear baseline periods lasting > 2 seconds.
- There is no clear hold period lasting > 2 seconds.

## Supplementary Information VI – Details of Source Estimation Techniques

Source inversion of sensor-level data involves constructing forward models to simulate the propagation of neural activity from the source to the sensor. For EEG data, we used a template boundary element model with sensor locations set according to the 10-10 system. For OPM data, sensor positions were derived from custom head cast models aligned to the subject’s structural MRIs. For the two subjects lacking MRIs, sensors were transformed to a template space using an iterative closest point algorithm to match the 3D scanned scalp to the template model. OPM head models used the single shell method^103^. To facilitate group-level statistical comparisons, a nonlinear warping was applied to align the anatomical scans.

Subject level common filters were computed from data concatenated across the multiple epochs of the experiment. Covariance matrices were computed in two frequency bands: tremor (peak postural tremor frequency ±1.5 Hz) and wide band beta (14-30 Hz). Each covariance matrix was truncated by its effective rank^104^. A covariance regularization of 1% was used.

## Supplementary Information VII – Implementation of Time Frequency Principal Component Analysis

tfPCA was applied to the group-averaged data with spectrograms concatenated for each motor epoch (time x four epochs). To avoid low frequency bias due to 1/f structure of EEG/OPM, spectra were log scaled. Spectrograms were also Z-normalized per subject to remove differences in SNR. We then constructed an *n × p* matrix ***X*** ([*n*: concatenated time] x [*p*: channel x frequency]) to which we applied PCA to yield coefficients ***V*** (*p* x number of components) for each spatio-spectral component, and latent scores ***Z*** (component x time). PCA coefficients were rotated using the Varimax algorithm to aid interpretability. We then projected components from the single trial level data *D_t_* (to compare experimental conditions and perform regressions) by computing *Z_t_ =* ***V****D_t_*. We also reconstructed components in the time frequency domain by multiplying trial-level data with the projection matrix ***P = VV^T^***, which we used for CNN tremor prediction analyses. In this way, the coefficients derived from the group averaged EEG data were used to project both EEG and OPM data to single trial level components.

## Supplementary Information VIII– Comparison of Source Localized Power in OPM and EEG Data

We provide a supplementary analysis comparing the source resolved changes in oscillatory power in the beta band between EEG and OPM data modalities. To do this, we provide an analysis of the full width at half maximum (FWHM). This is computed by searching for a subject specific maximum in the source resolved power change images, that lies within a 16mm radius of an ROI in the contralateral motor cortex (MNI: [-37 -25 62]). The FWHM was computed for each of the three axial planes of reference, and then averaged. A smaller FWHM indicates a steeper falloff. We also examined effect sizes obtained, comparing the average and range of t-statistics measured when contrasting with a baseline (see main text). Differences between OPM and EEG images were compared using t-statistics. The results are shown below.


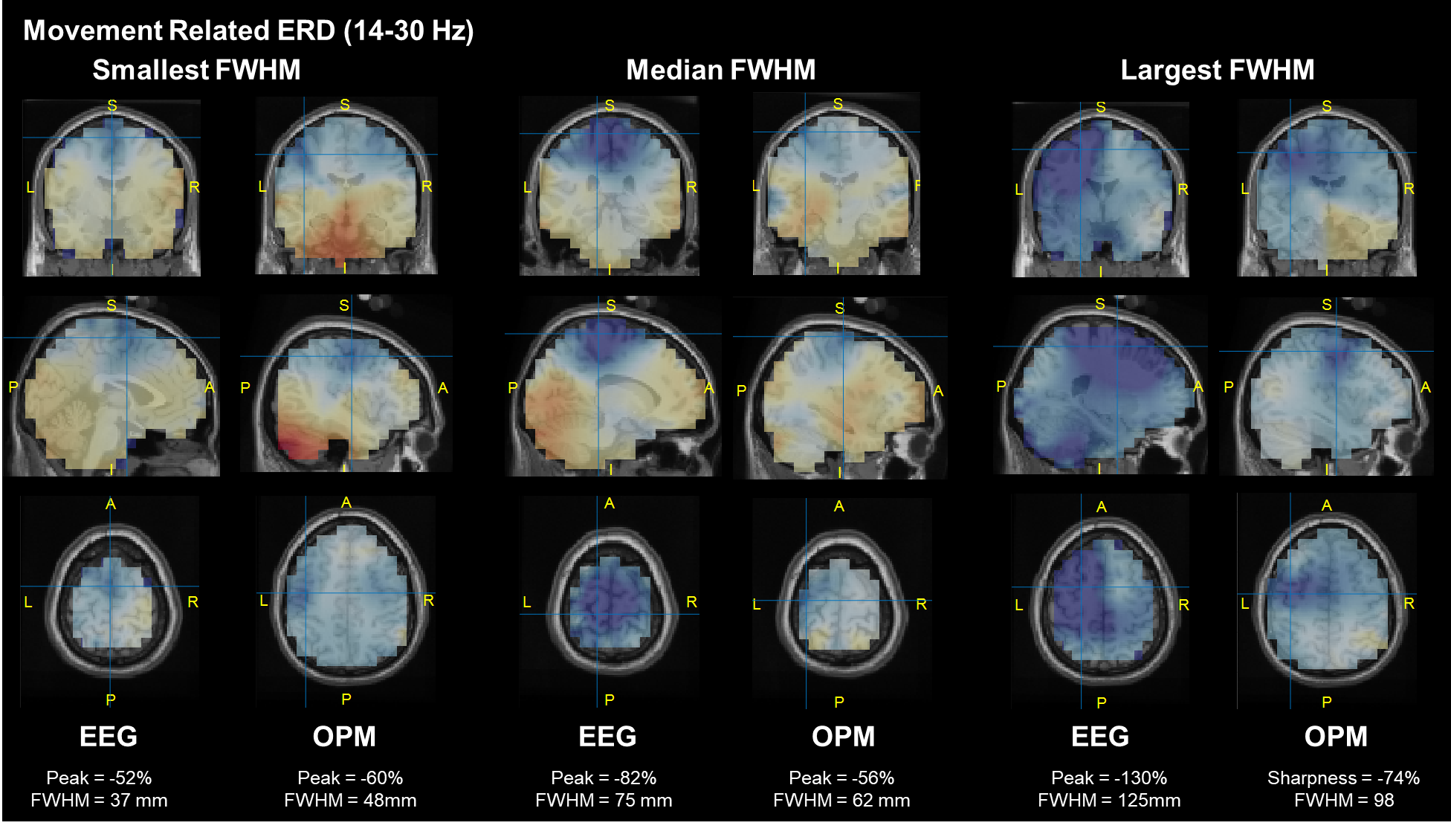


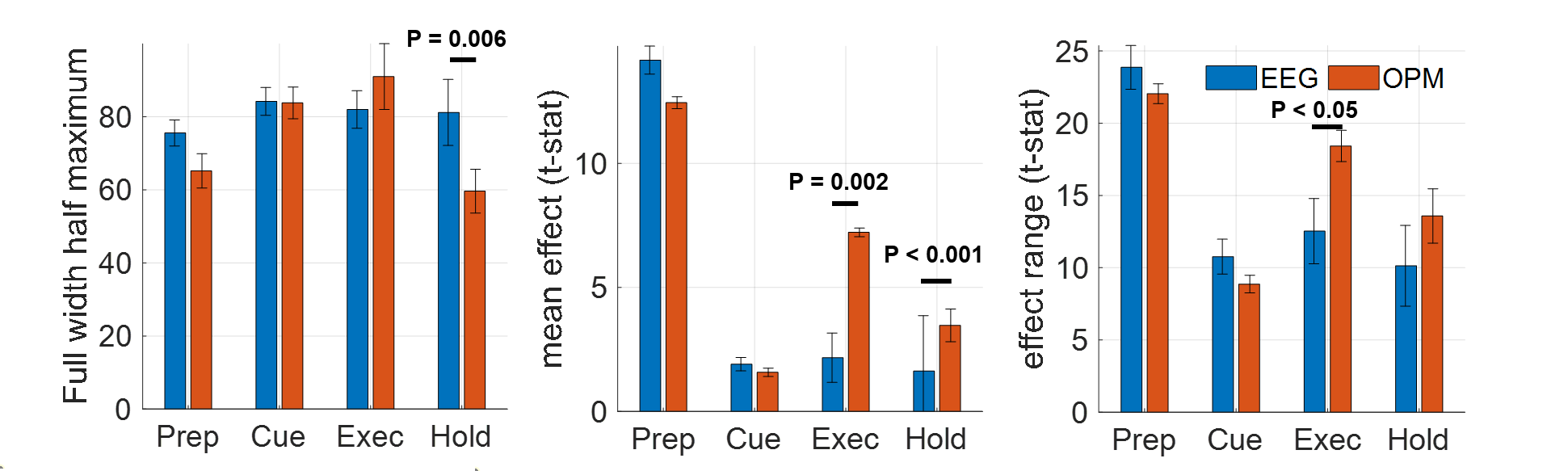


EEG and OPM source images were broadly comparable in quality. Sharper images were obtained using OPM during the HOLD periods (P = 0.006). OPM effect sizes were also significantly larger during low SNR periods, particularly at periods involving large reaching movements during execution and hold.

## Supplementary Information IX – Details of 3^rd^ Party Toolboxes

| Toolbox Name | Author | Year | Source/Reference |
| --- | --- | --- | --- |
| boundedline-pkg | Kelly Kearney | 2015 | <https://github.com/kakearney/boundedline-pkg> |
| brewermap | Stephen Cobeldick | 2014 | <https://github.com/DrosteEffect/BrewerMap> |
| Fieldtrip | Donders Institute, Radbound University | 2020 | <https://www.fieldtriptoolbox.org/> |
| linspecer | Jonathan C. Lansey | 2015 | <https://github.com/davidkun/linspecer> |
| Matlab Toolbox for Dimensionality Reduction (v0.8.1b) | Laurens van der Maaten | 2013 | <https://lvdmaaten.github.io/drtoolbox/> |
| splitvec | Bruno Luong | 2009 | [ttps://uk.mathworks.com/matlabcentral/fileexchange/24255-splitvec](https://uk.mathworks.com/matlabcentral/fileexchange/24255-splitvec) |
| SPM 12 | Wellcome Centre for Human Neuroimaging, University College London | 2020 | <https://www.fil.ion.ucl.ac.uk/spm/> |
